# Electrokinetically-Driven Assembly of Gold Colloids into Nanostructures for Surface-Enhanced Raman Scattering

**DOI:** 10.3390/nano10040661

**Published:** 2020-04-02

**Authors:** Hannah Dies, Adam Bottomley, Danielle Lilly Nicholls, Kevin Stamplecoskie, Carlos Escobedo, Aristides Docoslis

**Affiliations:** 1 Department of Chemical Engineering, Queen’s University, Kingston, ON K7L 3N6, Canada; h.dies@queensu.ca (H.D.); ce32@queensu.ca (C.E.); 2 Department of Chemistry, Queen’s University, Kingston, ON K7L 3N6, Canada; adam.bottomley@protonmail.com (A.B.); kevin.stamplecoskie@queensu.ca (K.S.); 3 School of Medicine, University of Toronto, Toronto, ON M5S 1A8, Canada; lilly.nicholls@mail.utoronto.ca

**Keywords:** metallic nanostructures, nanoparticles, electrokinetics, microelectrodes, surface-enhanced Raman scattering

## Abstract

Surface-enhanced Raman scattering (SERS) enables the highly sensitive detection of (bio)chemical analytes in fluid samples; however, its application requires nanostructured gold/silver substrates, which presents a significant technical challenge. Here, we develop and apply a novel method for producing gold nanostructures for SERS application via the alternating current (AC) electrokinetic assembly of gold nanoparticles into two intricate and frequency-dependent structures: (1) nanowires, and (2) branched “nanotrees”, that create extended sensing surfaces. We find that the growth of these nanostructures depends strongly on the parameters of the applied AC electric field (frequency and voltage) and ionic composition, specifically the electrical conductivity of the fluid. We demonstrate the sensing capabilities of these gold nanostructures via the chemical detection of rhodamine 6G, a Raman dye, and thiram, a toxic pesticide. Finally, we demonstrate how these SERS-active nanostructures can also be used as a concentration amplification device that can electrokinetically attract and specifically capture an analyte (here, streptavidin) onto the detection site.

## 1. Introduction

There is a global need for more effective sensors to detect (bio)chemical molecules in fluid systems. Raman spectroscopy has numerous advantages in this regard: it is molecularly specific, transparent to water, and can be performed in situ through the use of portable Raman spectrometers [1]. However, Raman scattering is inherently very weak. In order for the technique to be useful in the analysis of dilute solutions, Raman signals must be enhanced. One option is surface-enhanced Raman scattering (SERS), a method that can amplify Raman signals by many orders of magnitude through coherent oscillations of surface electrons on nanostructured surfaces (localized surface plasmons) and charge transfer or chemical enhancement [2,3].

A principal challenge in implementing SERS is in the fabrication of the nanostructured gold/silver surfaces required for the SERS activity. Often, patterning these surfaces requires expensive and laborious techniques such as electron-beam lithography or focused ion beam milling [4]. Recently, we have published work involving a method for fabricating nanostructured silver surfaces via the electric field-driven assembly of silver nanoparticles from colloidal suspension [5]. These surfaces were applied for the sensitive detection of relevant chemical analytes [6,7].

Although silver has high energy surface plasmon oscillations that can enable strongly enhancing substrates, it is also prone to oxidation, which greatly reduces the SERS enhancement, thereby limiting the long-term applicability of substrates [8,9,10,11]. Gold, as an alternative, has a lower enhancement but is less reactive, enabling more temporally-stable SERS surfaces. It also has well-known surface chemistry, allowing for the functionalization of surfaces with biological molecules to permit specific bio-sensing [9,10]. Previous groups have investigated the assembly of gold nanoparticles from microscale or nanoscale electrodes, for the purposes of forming nanowires for nanoscale electronic devices [11,12,13,14,15]. Other groups have used gold nanoparticles deposited onto solid substrates [16,17], or in colloidal suspension, directly [18], for SERS detection. However, in these examples, the ionic composition of the fluid matrix is not considered. This is an important consideration, as the ionic composition of the fluid matrix significantly affects the electrical double layer, which is subsequently expected to affect the intraparticle electrostatic forces, the dielectrophoretic forces on individual particles, and the AC electrokinetic fluid flows in the microelectrode system. 

In this work, we demonstrate a successful methodology for the template-free assembly of SERS-active gold nanostructures from colloidal gold with the aid of a spatially non-uniform electric field. We explore a range of conditions (electric field frequency, voltage, and medium conductivity) that enable the growth of such structures and investigate, specifically, the role of medium ionic composition on nanostructure growth. The SERS activity of these nanostructures is illustrated with the sensitive detection of rhodamine 6G and thiram, a toxic pesticide. Moreover, we showcase a major advantage and unique feature of these nanostructures, namely their ability to simultaneously function as concentration amplification devices that can electrokinetically drive the transport of analytes from the bulk onto the detection surface.

## 2. Materials and Methods 

### 2.1. Materials

Trichloro(1H, 1H, 2H, 2H-perfluorooctyl)silane, rhodamine 6G (R6G, 99%), thiram (Pestanal^®^, analytical standard), Cy3-Streptavidin, cysteamine, sodium citrate tribasic dihydrate, sodium sulfate, sodium chloride, gold (III) chloride trihydrate, and biotin-NHS (water-soluble) were purchased from Sigma Aldrich (Oakville, ON, Canada). Polished silicon wafers (4’’ in diameter) with a thermally grown SiO_2_ layer (0.5 μm) were purchased from University Water (South Boston, MA, USA). Millipore^®^ water (18.2 MΩ·cm) was used throughout the experiments. Reactant-free gold nanoparticles (50 nm, 1 OD, stabilized in 0.1 mM phosphate-buffered saline) were obtained from Cytodiagnostics, Inc. (Burlington, ON, USA). 

### 2.2. Gold Nanoparticle Preparation

Gold nanoparticles were prepared through the Turkevich method as described in Wuithschick et al. [19]. Specifically, 250 mL of 1 mM gold (III) chloride trihydrate was brought to a boil under continuous stirring. Then, 25 mL of 38.8 mM sodium citrate was added all at once. The solution immediately went from yellow to clear, then to dark blue/purple, then to a deep red, and was heated for 10 more minutes. After that it was allowed to stir for ~15 min without heat and finally quenched in an ice bath. No post-synthesis washing or purification was performed. The nanoparticle size distribution was characterized with a Hitachi H-7000 transmission electron microscope (Hitachi, Schaumburg, IL, USA). 

### 2.3. Conductivity Experiments

Sodium sulfate and sodium chloride were independently added to the Cytodiagnostics AuNPs and in-house prepared AuNPs to adjust the medium conductivity. The conductivity and particle Zeta potential were measured with a Zetasizer Nano ZS (Malvern Panalytical, Malvern, UK) with the applied potential adjusted to 0.5 V. 

### 2.4. Microchip Fabrication

The microfabrication of the electrodes was carried out at Nanofabrication Kingston (NFK, Innovation Park, Kingston, ON, Canada) via photolithography on silicon wafers. The microelectrode platform used has gold contact pads extending to circular tips with an adjacent tip-to-tip minimum gap of 40 μm. The Neutronix Quintel (NxQ4006) mask aligner was used to transfer the microelectrode pattern to the negative photoresist SU-8. Metals were deposited via electron beam evaporation: a 5 nm layer of chrome was used to improve the adhesion of the deposited Au layer (100 nm thickness, for the microelectrodes) to the silicon wafer. 

### 2.5. Chip Modification and Nanoparticle Deposition

The microelectrode surfaces were modified via surface silanization for better hydrophobicity (this improved droplet retention and promoted more extensive nanostructure assembly). The silanization procedure involved 3 min of oxygen plasma cleaning, followed by surface modification in a vacuum dessicator with 30 μL of trichloro(1H, 1H, 2H, 2H-perfluorooctyl)silane for 8 h. 

For nanoparticle deposition, a 5 μL sample of AuNP colloidal suspension was placed over the microelectrode gap, and the microelectrodes were activated at an AC frequency of 1 kHz–1 MHz, and a voltage of 3–7 V (amplitude). The nanoparticle assembly was run for 12 min. Following the assembly, the chips were rinsed with water and dried in a stream of air. Following SERS measurements, the surface was cleaned by gentle brushing with a cotton swab and a solution of dish soap and water. This regenerated the clean microelectrodes for future SERS substrate preparation. To ensure the reproducibility of the conductivity trends, 5 substrate depositions were completed at each conductivity value. 

### 2.6. Surface Characterization

Scanning electron microscopy (SEM) was performed at the Queen’s Facility for Isotope Research, on an MLA 650 FEG environmental SEM (FEI, Hilsboro, OR, USA), at a voltage of 5.00 kV. For the tilted SEM images, the platform was tilted by 10 degrees from the horizontal. Fluorescence microscopy was performed on an Olympus BX fixed stage microscope with a Cy3 filter (Olympus, Richmond Hill, ON, Canada). 

### 2.7. Biotin-Streptavidin Assay 

For the biotin-streptavidin-CY3 functionalization, after nanostructure assembly, the substrates were plasma-cleaned for 3 min. The substrates were then incubated in a 5.18 mM cysteamine solution in water for 72 h. The substrates were then rinsed with water for 30 s and dried in a stream of air. The substrates were then placed in a 24.56 mM biotin-NHS (water-soluble) solution in water for 1 h to allow biotin functionalization. The biotin-modified nanostructures were rinsed with water for 30 s and dried in a stream of air. Next, active capture was performed by reapplying the electric field in the presence of the 250 ppm streptavidin-Cy3 solution (in water) in order to concentrate the protein to the detection surface. The electric field was applied at a frequency of 10 kHz and a peak-to-peak voltage of 15 V. An 8 μL droplet of the 250 ppm streptavidin-Cy3 solution dissolved in water was micropipetted onto the center of the chip, and the field was applied for 15 min. The chips were then rinsed with water for 30 s and dried in a stream of air. Control experiments were performed by repeating the above procedure without the introduction of the biotin-NHS. 

### 2.8. Chemical Detection: Dropcasting 

Rhodamine 6G and thiram were dissolved in methanol and acetone, respectively. For their detection, 10 μL of analyte (10^−5^ M of R6G and 7 ppm of thiram) was dropcast upon the nanostructures, and the solvent was allowed to evaporate prior to SERS signal acquisition.

### 2.9. Raman Measurements and Spectral Processing

A HORIBA Jobin Yvon (Piscataway, NJ, USA) Raman Spectrometer (Model: LabRAM) with a 632.8 nm He/Ne laser (17 mW), 1800 L/mm grating, and an Olympus BX-41 microscope system were used. The collection of spectra was performed in the backscattered mode under the following conditions: ×100 microscope objective, 500 μm pinhole, 500 μm slit width, laser filter 10×, for a sampling time of 10 s (100 s for the thiram measurements), with 3 repeats. All Raman spectra were processed in MATLAB: they were background corrected through polynomial subtraction, and the noise was reduced with a Savitsky-Golay filter. The signal-to-noise ratio was calculated using the following equation:

(1) $$SNR=\frac{P}{\sqrt{MSE}}$$

where *P* is the height of the peak of interest (above *y_av_*) and *MSE* is the mean squared error of the spectrum, calculated via:

(2) $$MSE=\frac{1}{n}\overset{n}{\underset{1}{\sum }}{\left(y_{i}-y_{av}\right)}^{2}$$

where *n* is the number of points in the spectrum, *y_i_* are the individual Raman intensity values at each point, and *y_av_* is the average intensity of the spectrum. 

### 2.10. COMSOL Simulation

Simulations were performed on COMSOL Multiphysics (version 5.1). A cylindrical geometry (radius 270 μm, height 10 μm) was used for the simulations, with bipolar microelectrodes (electrode gap of 40 μm) used to simplify the electrode geometry (see Figure S1). The electrodes were simulated as gold with a thickness of 1 μm, upon an insulating SiO_2_ planar surface. Above the electrodes, we simulated an aqueous medium with varying conductivity. 

The electrical double layer was assumed to act as a capacitor, and the bulk fluid as a resistor. The electric domain was solved via the following partial differential equation:

(3) $$n\cdot \nabla V=\frac{i\omega C_{DL}}{\sigma_{m}}\left(V-V_{app}\right)$$

where **n** is a unit normal vector, $\sigma_{m}$ is the fluid (medium) conductivity, ω is the angular AC frequency, $C_{DL}$ is the capacitance per unit area of the electrical double layer, and $V_{app}$ is the electric potential applied to the microelectrodes. The capacitance of the double layer was approximated with a ratio of the dielectric constant to the Debye length, as described in Dies et al. [5,20].

The electrodes were simulated to be 180° out of phase, with applied voltages of +/− 7.5 V. The AC electroosmotic slip velocity was solved for via the following equation:

(4) vslip=12εmΛηmRe{(V−Vapp)Et*}

where $\varepsilon_{m}$ and $\eta_{m}$ are the permittivity and viscosity of the fluid medium (approximated in this simulation by the permittivity and viscosity of water) respectively, Λ is a correction factor (described in Dies et al. [5]), *V* is the potential outside the EDL, and $E_{t}^{*}$ is the complex conjugate of the component of the electric field tangential to the EDL [5,20].

## 3. Results

### 3.1. The Effect of AC Frequency on Gold Nanostructure Assembly

**Figure 1:**
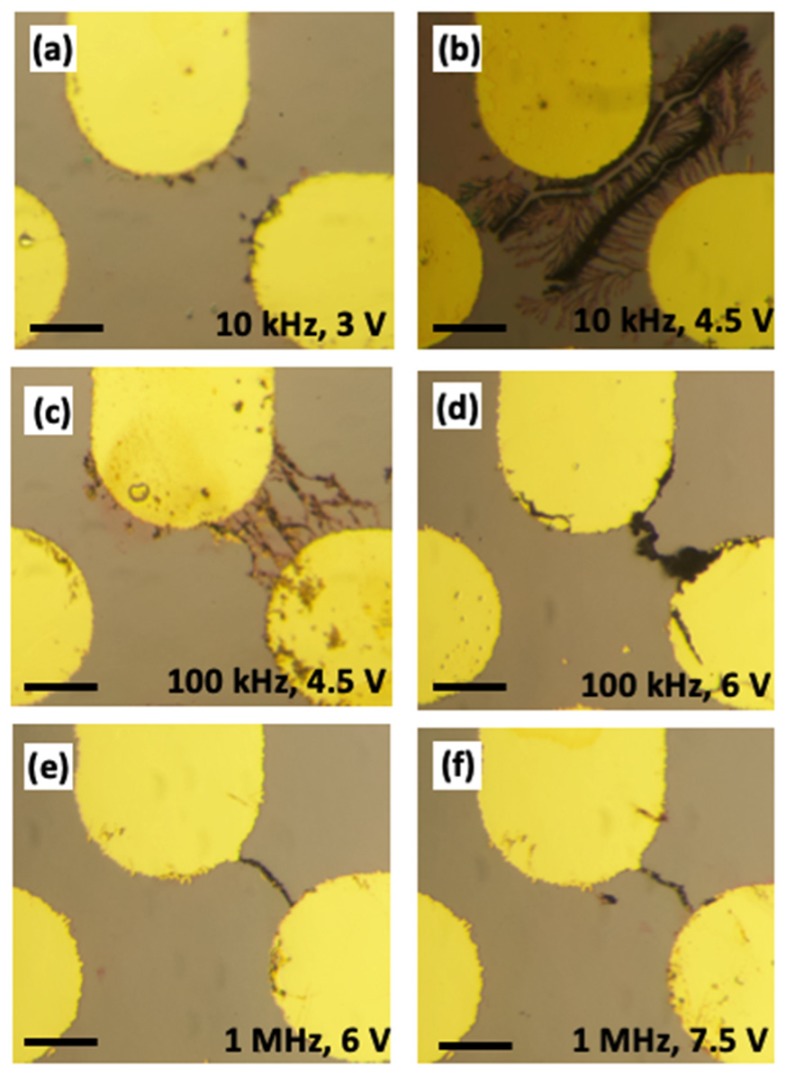
Gold nanostructures assembled at various electric field conditions. In all images, the top and right electrodes were activated with an AC potential. (**a**) 10 kHz, 3 V amplitude. (**b**) 10 kHz, 4.5 V amplitude. (**c**) 100 kHz, 4.5 V amplitude. (**d**) 100 kHz, 6 V amplitude. (**e**) 1 MHz, 6 V amplitude. (**f**) 1 MHz, 7.5 V amplitude. The scale bar represents 40 μm.

To generate the spatially non-uniform electric field, we employed a planar quadrupolar gold microelectrode array, photolithographically deposited on a polished oxidized silicon substrate (using chromium as the adhesion layer). Figure 1 shows the frequency and voltage-dependent morphology of the gold nanostructures formed when a droplet of colloidal gold nanoparticles is dispensed on top of the energized microelectrode array. For simplicity, only two of the four microelectrodes were activated each time (with 180° of phase separation in the AC electric field between adjacent electrodes), as described in Dies et al. [5]. The allowable voltage that can be applied across the microelectrode tips increases with frequency, as both damaging electrolytic reactions and replacement of the chrome adhesion layer occur more readily at lower frequencies. Any damage to the microelectrodes induced in the nanostructure assembly process limits the reusability of the platforms. We noticed that below 10 kHz, the electrodes were significantly damaged, and there was no nanostructure growth observed; therefore, we used 10 kHz as the lowest experimental frequency. As shown in Figure 1, we included experimental redundancy in the voltage and frequency, i.e., each frequency studied has a voltage that overlaps with a higher and a lower frequency setting, to isolate the individual effects of voltage and frequency on the nanostructure geometry. In Figure 1, the right column (Figure 1b,d,f) represents the maximum allowable non-damaging voltages at each frequency. We observed the growth of nanostructures into two main, frequency-dependent, structure types. At low frequencies (<100 kHz), the nanoparticles form branched nanostructures. When the frequency is increased (>100 kHz), the nanoparticles form structures as shown in Figure 1c–f). These structures form as nanowires, that grow from both electrodes towards each other, following electric field lines. No structures were observed to grow at frequencies below 1 kHz, or above 1 MHz. We noted that the nanowires shown in Figure 1d–f were the dynamic result of multiple nanowires coalescing throughout the deposition. The mutual dielectrophoretic force between these wires increases with voltage [21], potentially enabling the coalescence of multiple nanowires into a broader nanostructure.

**Figure 2:**
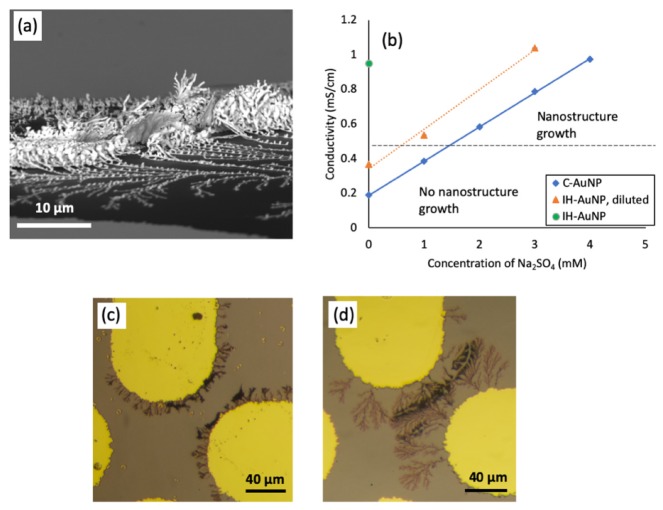
(**a**) A tilted SEM image of gold nanostructures grown at 10 kHz, 4.5 V. (**b**) The effects of medium conductivity (modulated via added sodium sulfate) on nanostructure growth (10 kHz, 4.5 V). Error bars are included on the conductivity values; all relative standard deviations were less than 1%. Points above the conductivity threshold (dashed line) resulted in nanostructure growth on the microelectrodes, whereas those below the dashed line resulted in no growth. (**c**) Cytodiagnostics AuNP nanostructures and (**d**) in-house AuNP nanostructures assembled above the conductivity threshold (10 kHz, 4.5 V).

The tilted SEM image shown in Figure 2a adds more information about the geometry of the nanostructures and provides insight into the growth mechanism of the latter. Notably, as shown in Figure 2a, the structures formed at 10 kHz have a significant growth away from the plane of the electrodes. We postulate that this out-of-plane growth occurs due to repulsive forces from the interacting nanostructures, as well as the influence of AC electroosmosis, as described in Section 3.3. Our group has work in preparation that suggests that metallic nanostructures have a positive free energy of interaction, that is, they spontaneously repel each other. Therefore, to avoid closely associating, the growing nanostructures buckle upwards away from the silicon substrate. 

### 3.2. The Effect of Medium Conductivity on Nanostructure Assembly 

The conductivity of the suspension has a significant effect on nanostructure growth, as was observed through an experiment in which we attempted to assemble the nanostructures with “Reactant-Free” gold nanoparticles from Cytodiagnostics, Inc (“C-AuNPs”). With these nanoparticles, we did not observe any nanostructure growth. Notably, the C-AuNPs are washed and extensively purified to be 99% free of residual ions from the nanoparticle preparation process. To assess the role of suspension conductivity, we added small amounts of a spectator ion, sodium sulfate, to the reactant-free nanoparticles. By increasing their conductivity above a threshold value, as shown in Figure 2b, we imparted the ability to grow nanostructures at 10 kHz, 4.5 V amplitude (see Figure 2c). To confirm the effects of conductivity, and to assure that these results were not due to other differences in the media between these two nanoparticle types, we performed the same experiment with the in-house prepared AuNPs (“IH-AuNPs”). We diluted the IH-AuNPs 3.33 times such that they had a similar optical density and medium conductivity to the C-AuNPs. At this conductivity, no nanostructure growth was observed. Next, we added small amounts of sodium sulfate, and again we observed that at a certain threshold conductivity, the AuNP solutions recovered the ability to grow nanostructures (see Figure 2d). 

To investigate the role of the ion identity, we repeated the aforementioned conductivity experiment with sodium chloride rather than sodium sulfate. The results of this experiment are shown in the Supplementary Information (Figure S2). Notably, we determined that NaCl was able to “rescue” the diluted IH-AuNP suspension, but not the C-AuNP suspension. That is, above the conductivity threshold illustrated in Figure 2b, the IH-AuNP suspensions were capable of nanostructure assembly; however, the C-AuNP suspensions were still incapable of nanostructure assembly. These results suggest that a threshold medium conductivity is necessary but not sufficient for nanostructure assembly, and encourage future research into the role of ion identity. 

While the addition of sodium sulfate above a threshold value “rescues” both the C-Au NPs and the IH-Au NPs, there is still an apparent difference in the morphology of the nanostructures that grow from these two Au NP suspensions. We characterized the NPs in order to assess whether the size or surface charge could be impacting the growth. The zeta potentials of the nanoparticles were the following: IH-Au NPs −28.0 mV +/− 3.1 mV, and C-AuNPs −35.9 mV +/− 7.0 mV. The IH-Au NPs had a unimodal size distribution with an average diameter of 12.4 +/− 2.1 nm, whereas the C-Au NPs had a bimodal distribution with diameters of (1) 12.8 +/− 1.9 nm and (2) 53.4 +/− 7.9 nm. The particle size distribution histograms are shown in the Supplementary Information (Figure S3), and the TEM images used to determine these size distributions are shown in Figure S4. The larger size distribution in the C-Au NPs may have a limiting role in the nanostructure growth, preventing the intricate branching and extended nanostructure growth observed for the IH-Au NPs. 

Because we observed a clear morphology difference between the nanostructures grown from the C-Au NP suspensions and the IH-Au NP suspensions, we performed experiments in which we added small amounts (ranging from 1 μM to 100 μM) of gold (III) chloride trihydrate to C-Au NP suspensions above the established conductivity threshold, to determine if this was a key and necessary missing component of the C-Au NP suspension. In the lower molarity regime (1 μM), no difference in nanostructure growth was observed, and in the intermediate molarity regime (10 μM), significant damage to the microelectrodes was observed. Above 10 μM, the nanoparticles precipitated upon addition of the gold salt. These results are summarized in the supplementary information (Table S1).

Notably, the nanostructure branching at lower frequencies has morphologic similarity to the dendritic structures formed by electrochemical deposition at low frequencies and applied voltages. Numerous groups have investigated these structures and have used the model of diffusion limited aggregation to explain their growth [22,23]. It is possible that a similar mechanism controls the growth (local to the microelectrodes) of both types of structures, with the building blocks representing nanoparticles in the mechanism described here, and solvated ions in electrochemical growth. There may even be a contribution from dissolved metallic ions in solution (other than their effects on conductivity as discussed above) in the growth of the nanostructures described here. 

### 3.3. Simulation and Discussion of the Phenomenon of Nanostructure Growth

In this section, we discuss our observations and attempt to correlate the nanostructure formation characteristics with the AC frequency of the applied electric field and resulting electrohydrodynamic effects. It is well known that the motion and interactions of colloidal particles inside an alternating current (AC) electric field can be influenced by a suite of electrokinetic effects, including direct forces on constituent nanoparticles, as well as bulk electrohydrodynamic flows. A primary force involved in nanoparticle assembly is dielectrophoresis (DEP), which acts directly on induced dipoles in a non-uniform electric field [24]. For spherical particles; the dielectrophoretic force, *F_DEP_*; is given by the following equation:

(5) F→DEP=2πr3Re(K˜(ω)∇|E→|2

where *r* is the radius of the nanoparticle; $\tilde{K}$ is the complex-valued, frequency-dependent Clausius-Mossotti factor; ω is the frequency; and $\vec{E}$ is the electric field intensity [24]. 

For metallic nanoparticles, the Clausius-Mossotti factor is 1 at practically all experimentally relevant frequencies; therefore, the dielectrophoretic force is positive and in the direction of the electric field gradient. Notably, it is also strongest at regions of high electric field gradient. In our microelectrode system, this corresponds to the microelectrode edges [5]. Additional contributors to the nanoparticle dynamics include electrohydrodynamic flows, including AC electroosmosis and AC electrothermal flow [25]. AC electroosmosis results from the action of a tangential electric field on the electrical double layer. It is a frequency-dependent flow, and results in bulk fluid flow towards the microelectrode gap and across the surface of the microelectrodes (see Figure S1). The frequency dependence of AC electroosmosis is largely related to the charging time of the electrical double layer. Below the charging frequency of the double layer, the electric double layer has time to completely form, therefore comprehensively shielding the AC potential [21]. With no applied potential, the tangential electric field and, therefore, the AC electroosmotic flow are negligible. At frequencies much greater than the charging frequency, the double layer does not have time to form, and therefore there is insufficient charge to establish the AC electroosmotic flow. There is, therefore, an intermediate frequency at which the double layer has time to form, yet does not completely shield the electric potential, and the AC electroosmotic flow is maximized. In the case of electrokinetic nanoparticle assembly, it is foreseeable that there is also an optimal AC electroosmotic flow (i.e., not the maximum flow). This flow would permit nanoparticle sampling via convective fluid flow from the bulk to the surface of the microelectrodes, yet would not override the ability to perform dielectrophoretic manipulation of the nanoparticles. 

**Figure 3:**
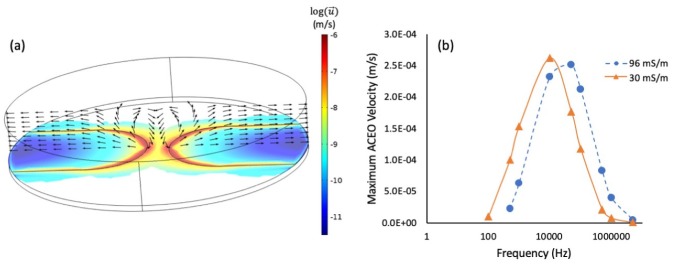
COMSOL simulation of AC electroosmotic flow. (**a**) Simulation results at 100 kHz, 30 mS/m medium conductivity. The image has been tilted for visualization. Bipolar electrodes with the same interelectrode spacing (40 μm) were chosen for simplicity. In the plane of the microelectrodes, the color legend indicates the magnitude of the electroosmotic velocity. The arrows perpendicular to the electrode plane indicate the direction of the AC electroosmotic velocity. (**b**) The maximum AC electroosmotic velocity at varying AC frequency and medium conductivity. Each point in these curves represents the maximum AC electroosmotic velocity obtained from a single independent COMSOL simulation. The conductivity of the IH-AuNPs is approximately 96 mS/m (above the established conductivity threshold), and 30 mS/m approximates that of the C-AuNPs (below the established conductivity threshold).

To investigate its influence on nanostructure assembly, we performed a COMSOL simulation of AC electroosmoosis for our microelectrode system geometry. The simulation design is described in Dies et al. [5], and the results are shown in Figure 3. Briefly, we simulate the electric domain (potential and electric field) with the electrical double layer. Then, we include the electroosmotic slip velocity in the simulation using the Helmholtz-Smoluchowski approximation [20]. The flow, as shown in Figure 3a, moves in a direction from the fluid bulk towards the surface of the microelectrodes, with the fluid circulation acting to supply material to the growing nanostructures. We also performed independent simulations of the maximum AC electroosmotic velocity at seven different frequencies, ranging from 100 Hz–10 MHz, and at two different conductivities: 30 mS/m and 96 mS/m (Figure 3b). The peak electroosmotic velocity becomes smaller and also occurs at higher AC frequencies as conductivity increases. Importantly, we notice that for our experimental conditions, the AC electroosmotic velocity peaks between 10–100 kHz, in the region where we see the most significant nanostructure growth. Our experimental observations show that the most substantial nanostructures grow in a regime where AC electroosmotic flow contributes significantly. Above 1 MHz, this effect is reduced in two ways: (1) AC electroosmosis decreases in magnitude; and (2) AC electrothermal flow starts to become a dominant flow, acting in the opposite direction of AC electroosmosis and counteracting the associated nanomaterial supply [26]. 

Finally, other groups have observed and simulated the phenomenon of decreased branching at higher AC electrokinetic frequencies and voltages [27,28]. Liu et al. [27] used an RC circuit to model a similar system, and determined that at 10 kHz, induced charge electroosmosis (ICEO) has a location-specific force vector that initially draws nanoparticles from the fluid bulk towards the electrode tip, and then in closer proximity to the growing nanoparticle structure, becomes a repulsive force that directs nanoparticles upwards in the direction of the fluid bulk. This is similar to the direction of the AC electroosmosis that we determine from our model, as shown in Figure 3a. Above 10 kHz, and at higher voltages, the AC electroosmotic force decreases (as shown in Figure 3b), and the DEP force (which scales as *V*^2^ [21]) is able to overcome this force, resulting in nanowires that extend across the microelectrode gap.

### 3.4. SERS Activity of the Nanostructures

**Figure 4:**
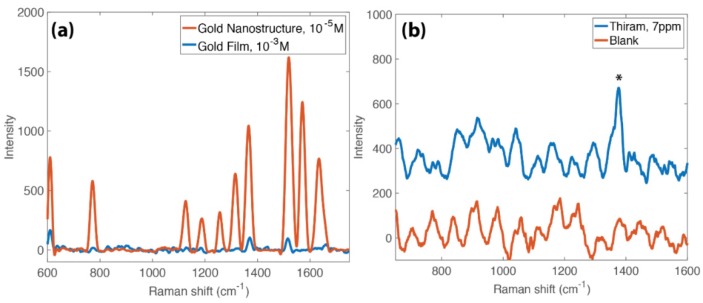
Chemical detection on the nanostructures. (**a**) Rhodamine 6G Raman spectra on a gold nanostructure (red) and a gold film (blue). These spectra are used to determine the surface enhanced Raman scattering (SERS) enhancement factor of 1.5 × 10^3^. (**b**) Chemical detection of thiram on the nanostructures at a concentration of 7 ppm. The key peak at 1385 cm^−1^ is identified with an asterisk. Note that the oscillatory background in part (**b**) is an artifact of the notch filter.

Both the ‘nanotrees’ and the ‘nanowires’ were observed to cause SERS enhancement. For the remainder of the results shown, we focused on the ‘nanotrees’ (deposited at 10 kHz, 4.5 V amplitude) due to their increased surface area for analyte adsorption (relative to the nanowires). Figure 4a shows the results from SERS measurements of R6G, a Raman dye commonly used to characterize SERS substrates. By comparing these spectra with those of plain gold film, we obtain a SERS enhancement factor (EF) of 1.5 × 10^3^. This value is certainly lower than the EF enabled by silver nanostructures [5], and is also lower than some EFs obtained by some research groups producing gold-based SERS substrates. For example, Fusco et al. report an enhancement factor for their substrates (produced via the self-assembly of Au nanoparticle aerosols) of 10^7^–10^8^ [29], Kundu and Jayachandran report an EF of 10^5^–10^6^ for their DNA templated-nanowires [30], and Huang et al. report an enhancement factor of 2 × 10^4^ for their dendritic gold nanostructures [31]. However, our nanostructures are relatively simple to prepare; they yield an EF comparable to commercially available SERS substrates and sufficient to enable the analysis of dilute suspensions. Figure 4b shows the results of the SERS detection of thiram, a pesticide used in fruit and soybean farming, that is hepatotoxic upon metabolization. We dropcast thiram on the surface of the nanostructures at 7 ppm (2.5 × 10^−5^ M), the United States Environmental Protection Agency maximum residue limit [32], and were able to clearly identify the key peak at 1385 cm^−1^. The peak is identifiable from the background noise by a peak signal to noise ratio of 4.48. Unprocessed (no background removal, no signal processing) data are available in the Supplementary Information (see Figure S6). We acknowledge that the SERS substrates produced in this work are inhomogeneous in terms of surface coverage, and, therefore, the SERS intensity would be expected to vary correspondingly. All SERS measurements displayed here were taken directly between the two microelectrodes, and on nanostructures visualized via SERS microscopy. Away from the microelectrodes, where no deposition is observed, the Raman intensity is very low. In future work, it would be helpful to characterize this further with surface mapping of the SERS intensity. 

### 3.5. Nanostructure Functionalization

A major advantage of using gold as a substrate for sensing is its ability to be functionalized, enabling the attachment of biological molecules and the potential for specific biosensing. We performed a biotin-streptavidin assay on these nanostructures to assess this potential. The gold was functionalized with cysteamine via its thiol group, followed by the oriented attachment of biotin-NHS to the cysteamine (as described in [33,34]). This platform was used for an assay of fluorescently-labeled (Cy3) streptavidin. For visualization of this experiment, we performed this assay on plain gold films (see Figure S5), as well as on the gold nanostructures.

### 3.6. Concentration Amplification Effect

**Figure 5:**
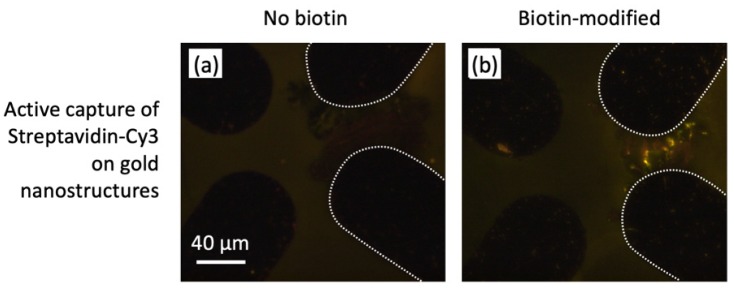
Fluorescence results from the biotin-streptavidin Cy3 assay. White dashed lines indicate the microelectrodes that were used to assemble the nanostructures, as well as, for active capture, the microelectrodes that were activated for analyte collection. (**a**) Active capture (analyte collected at 10 kHz, 15 V peak-to-peak, for 15 min) of streptavidin-Cy3 on a non-biotinylated gold nanostructure (nanotrees). (**b**) Active capture (analyte collected at 10 kHz, 15 V peak-to-peak, for 15 min) of streptavidin-Cy3 on a biotinylated gold nanostructure (nanotrees).

One way to improve the detection performance of a surface-based sensing device, where analyte transport is diffusion-limited, is by amplifying the surface concentration of the analyte. Here we perform a biological assay on the surface of the gold nanostructures and illustrate how concentration amplification can be achieved by using an “active capture” method. The Au nanostructures constitute physical extensions of the planar microelectrodes, i.e., when energized with an AC electrical signal, they can bring about strong electrokinetic effects (predominantly AC electroosmosis and dielectrophoresis), which in turn can transport and capture the analyte onto the SERS detection site. With the “active capture” method, we observed significant fluorescence on the biotinylated gold nanostructures, and not on the non-biotinylated structures (Figure 5a,b). To the best of our knowledge, this is the first demonstration of active electrokinetic capture onto SERS substrates combined with a biological surface modification that imparts analyte specificity. This work provides a platform for extending this nanostructure functionalization technique to more SERS-active analyte-antibody pairs, such as cocaine detection with anti-cocaine functionalization.

## 4. Conclusions

In conclusion, we have developed a method for the AC electrokinetic assembly of gold nanoparticles into SERS-active nanostructures and demonstrated its application to biological and chemical sensing. We found that the nanostructure growth displays a strong dependency on the AC frequency, AC potential, and medium conductivity. We have determined for the first time a threshold conductivity for nanoparticle association and electrokinetic assembly into microscale structures. This threshold conductivity appears to be necessary, but not sufficient, encouraging further study into this previously overlooked contributing variable. These nanostructures are applied with an active electrokinetic concentration mechanism combined with substrate functionalization for the specific retention of streptavidin-Cy3, and are also applied for the sensitive chemical detection of rhodamine 6G and thiram. 

## Supplementary Materials

The following are available online at https://www.mdpi.com/2079-4991/10/4/661/s1, Figure S1: The direction of alternating current electroosmosis (ACEO) above the microelectrode surface; Figure S2: Nanostructure assembly with the addition of NaCl; Figure S3: Nanoparticle size distribution histograms obtained from the TEM images; Figure S4: TEM images used for nanoparticle size distribution histograms; Figure S5: The fluorescence images for visualization of streptavidin-Cy3 capture; Figure S6: Supplementary data to the SERS spectra presented in Figure 4; Table S1: Results from the addition of gold salts to the C-AuNP suspensions. Both salt solutions were above the previously established conductivity threshold.
