# Supplementary material for: Electrokinetically-Driven Assembly of Gold Colloids into Nanostructures for Surface-Enhanced Raman Scattering
Source: Nanomaterials (Basel). 2020 Apr 2;10(4):661. doi: 10.3390/nano10040661 (PMC7221533; doi:10.3390/nano10040661)
Supplement: Supplementary file 1 [file nanomaterials-10-00661-s001.pdf]

Supplementary Material

Electrokinetically-Driven Assembly of Gold Colloids into Nanostructures for Surface-Enhanced Raman Scattering

Hannah Dies [1], Adam Bottomley [2], Danielle Lilly Nicholls [3], Kevin Stamplecoskie [2], Carlos Escobedo [1] and Aristides Docoslis [1,*]

[1] Department of Chemical Engineering, Queen's University, Kingston, ON, K7L 3N6, Canada; h.dies@queensu.ca (H.D.); ce32@queensu.ca (C.E.)
[2] Department of Chemistry, Queen's University, Kingston, ON, K7L 3N6, Canada; adam.bottomley@protonmail.com (A.B.); kevin.stamplecoskie@queensu.ca (K.S.)
[3] School of Medicine, University of Toronto, Toronto, ON, M5S 1A8, Canada; lilly.nicholls@mail.utoronto.ca
* Correspondence: docoslis@queensu.ca; Tel.: +01-(613)-533-6949

Received: 7 March 2020; Accepted: 31 March 2020; Published: date

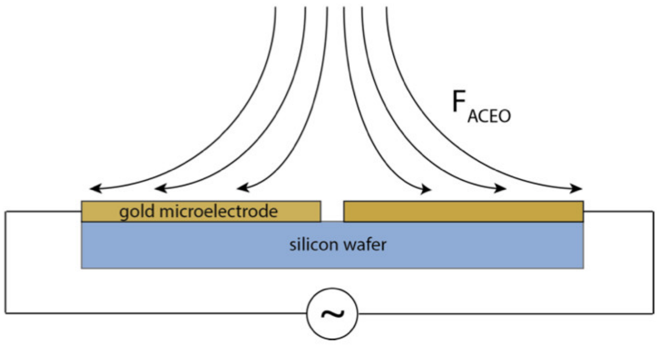

Figure S1. The direction of alternating current electroosmosis (ACEO) above the microelectrode surface. Schematic is not to scale.

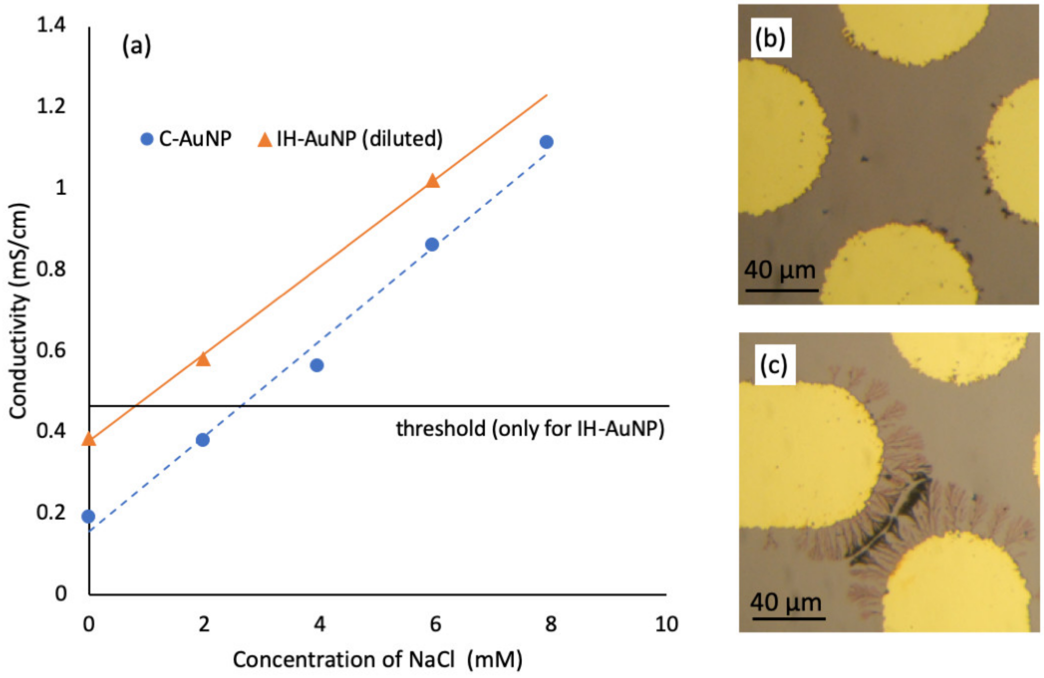

Figure S2. Nanostructure assembly with the addition of NaCl. (**a**) The NaCl addition was able to “rescue” the diluted in house Au NPs (IH-Au NPs); i.e. all points above the indicated threshold conductivity produced nanostructures. Conversely, the addition of NaCl to the Cytodiagnostics Au NPs (C-Au NPs) did not enable nanostructure growth at any experimental conductivities. (**b**) Results from attempted nanostructure assembly (at 10 kHz, 4.5 V amplitude) with C-Au NPs above the threshold conductivity. (**c**) Results from nanostructure assembly (at 10 kHz, 4.5 V amplitude) with IH-AuNPs above the threshold conductivity.

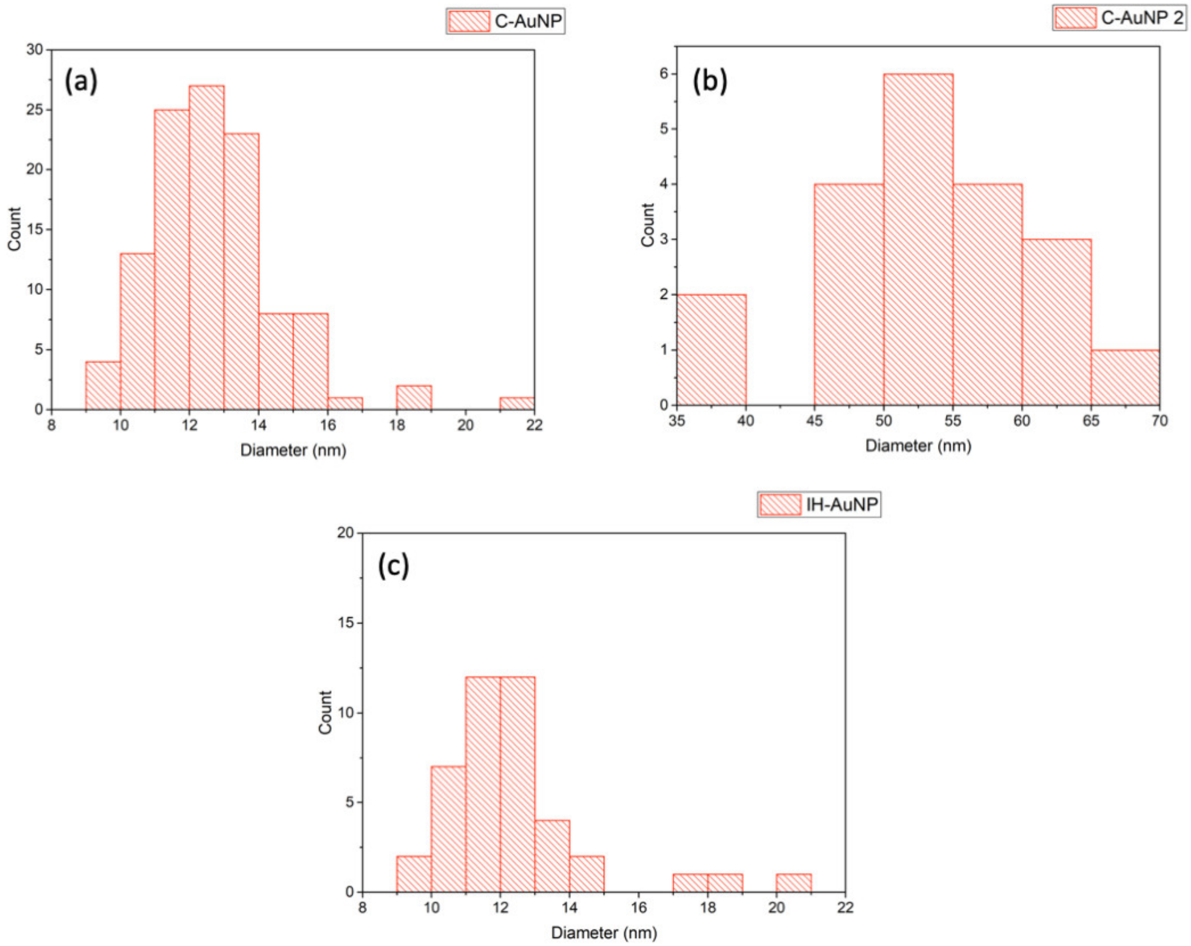

Figure S3. Nanoparticle size distribution histograms obtained from the TEM images (shown in Figure S4). (**a**) The Cytodiagnostics Au NP smaller diameter size distribution: average 12.8 +/− 1.9 nm. (**b**) The Cytodiagnostics Au NP larger diameter size distribution: average 53.4 +/− 7.9 nm. (**c**) The in-house prepared Au NP (unimodal) size distribution: 12.4 +/− 2.1 nm.

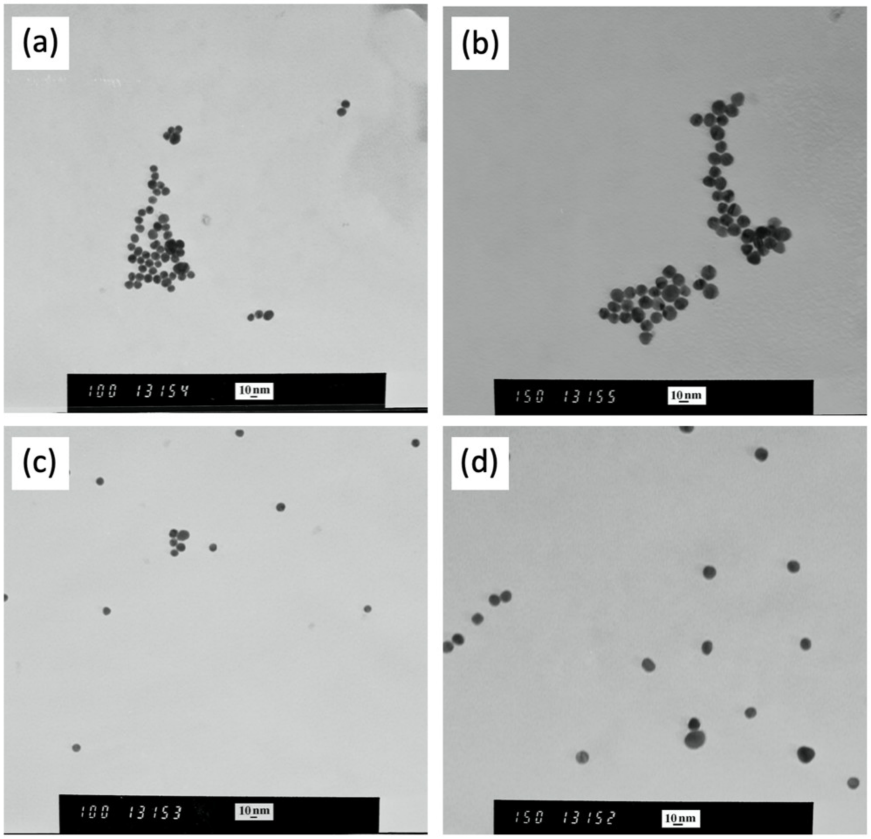

Figure S4. TEM images used for nanoparticle size distribution histograms. (**a**) and (**b**) are TEM images of the Cytodiagnostics Au NPs, and (**c**) and (**d**) are TEM images of the in-house prepared Au NPs.

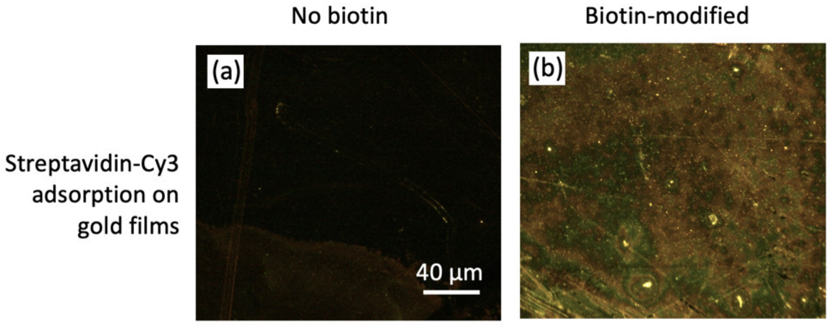

Figure S5. The fluorescence images for visualization of streptavidin-Cy3 capture. (**a**) Fluorescent image of a non-biotinylated (only cysteamine-coated) gold film. (**b**) Fluorescent image of a biotinylated gold film.

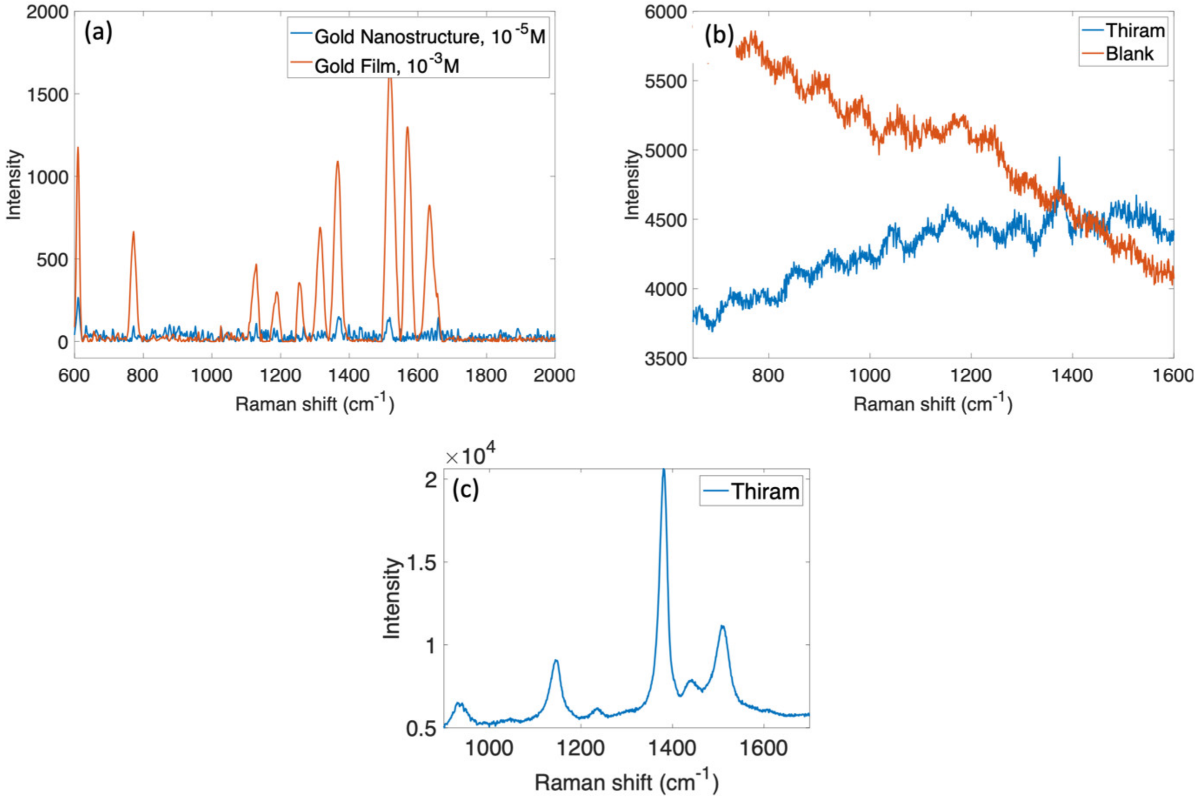

Figure S6: Supplementary data to the SERS spectra presented in Figure 4. (**a**) Unprocessed data for R6G on SERS substrates and gold films. This reading had an automatic background subtraction (polynomial), however no filtering (Savitsky-Golay) of the data was done. The filtered data is shown in Figure 4(**a**). (**b**) Unprocessed data (no background subtraction, no filtering) for thiram on SERS substrates and blank SERS substrates. The processed data is shown in Figure 4(**b**). (**c**) 100 ppm thiram on a gold SERS substrate.

Table S1. Results from the addition of gold salts to the C-AuNP suspensions. Both salt solutions were above the previously established conductivity threshold.

Salt Solution	Concentration of $HAuCl_4$	Results
6 mM NaCl	1 μM	No effect (did not grow)
	10 μM	Damaging to microelectrodes
	100 μM	Nanoparticles aggregated
3 mM Na_2SO_4	1 μM	No effect (grew as before, still no branching)
	10 μM	Nanoparticles aggregated
